# HTLV-1 reverse transcriptase homology model provides structural basis for sensitivity to existing nucleoside/nucleotide reverse transcriptase inhibitors

**DOI:** 10.1186/s12985-024-02288-z

**Published:** 2024-01-10

**Authors:** Nicolas Tardiota, Noushin Jaberolansar, Julia A. Lackenby, Keith J. Chappell, Jake S. O’Donnell

**Affiliations:** 1 The Australian Institute for Bioengineering and Nanotechnology, The University of Queensland, St Lucia, QLD 4072 Australia; 2 The School of Chemistry and Molecular Biosciences, The University of Queensland, St Lucia, QLD 4072 Australia; 3 Australian Infectious Disease Research Centre, The University of Queensland, St Lucia, QLD 4072 Australia

## Abstract

The human T-lymphotropic virus type 1 (HTLV-1) infects millions of people globally and is endemic to various resource-limited regions. Infections persist for life and are associated with increased susceptibility to opportunistic infections and severe diseases including adult T cell leukemia/lymphoma and HTLV-1-associated myelopathy-tropical spastic paraparesis. No HTLV-1-specific anti-retrovirals have been developed and it is unclear whether existing anti-retrovirals developed for treatment of human immunodeficiency virus (HIV) have efficacy against HTLV-1. To understand the structural basis for therapeutic binding, homology modelling and machine learning were used to develop a structural model of the HTLV-1 reverse transcriptase. With this, molecular docking experiments using a panel of FDA-approved inhibitors of viral reverse transcriptases to assess their capacity for binding, and in turn, inhibition. Importantly, nucleoside/nucleotide reverse transcriptase inhibitor but not non-nucleoside reverse transcriptase inhibitors were predicted to bind the HTLV-1 reverse transcriptase, with similar affinity to HIV-1 reverse transcriptase. By strengthening the rationale for clinical testing of therapies such as tenofovir alafenamide, zidovudine, lamivudine, and azvudine for treatment of HTLV-1, this study has demonstrated the power of in silico structural biology approaches in drug design and therapeutic testing.

**Supplementary Information:**

The online version contains supplementary material available at 10.1186/s12985-024-02288-z.

## Introduction

Once established, human T-lymphotropic virus type 1 (HTLV-1) retroviral infections usually persist for life. While less severe than the closely related Human Immunodeficiency Virus (HIV), HTLV-1 infections result in sub-clinical immune suppression and are associated with a higher relative risk (RR) of all-cause mortality (RR 1.57; 95% CI 1.37–1.80) and a range of serious sequelae throughout life. Most seriously, HTLV-1 causes adult T cell leukemia/lymphoma (ATLL), a rare and extremely aggressive peripheral T cell cancer in 5% of cases, and HTLV-1-associated myelopathy-tropical spastic paraparesis (HAM-TSP), a degenerative autoimmune disease of the peripheral nervous system in a further 5% of cases [1–3]. Although uncommon in many developed countries, HTLV-1 is estimated to infect 10 to 20 million individuals globally [1–3].

No specific therapies have been developed to prevent, manage, or cure HTLV-1 infections, other than allogenic hematopoietic stem cell transplantation; a high-risk therapy used in treatment of aggressive ATLL [4]. Instead, interventions have focused on the management of HTLV-1-associated diseases, with limited success [1–3]. Adopting a pragmatic approach, research efforts have focused on testing anti-retroviral therapies developed for HIV against HTLV-1 such as Zidovudine (3′-azido-3′-deoxythymidine), tenofovir (9-(R)-[2-(phosphonomethoxy)propyl] adenine, PMPA), and lamivudine (2,3-dideoxy-3-thiacytidine) [5–7]. For many such compounds, in vitro testing has been able to demonstrate a successful reduction in proviral load [5–9]. However, of the few HTLV-1-related clinical studies performed, anti-retroviral therapies have not achieved this effect among chronically infected individuals [10]. One explanation for this discrepancy is that the HTLV-1 proviral load during chronic infection is maintained by reverse transcriptase-independent clonal proliferation [11]. By contrast, throughout the acute phase of infection, reverse transcriptase-mediated infective spread predominates and is critical for the establishment of a chronic infection [11]. This has led to the suggestion that anti-retroviral therapies might be more likely to suppress or eliminate an HTLV-1 infection when used as either pre- or post-exposure prophylaxis, to reduce one’s risk of transmission, and as supportive therapy in the context of secondary disease; approaches which have been particularly effective for HIV prevention and management [12]. Among at-risk populations, conducting clinicals trial to assess the effectiveness of anti-retroviral therapy of reverse transcriptase inhibitors is possible; however, given that reporting of new HTLV-1 infections among adults is rare, the pool of patients available for inclusion in any trial is likely to be small. Therefore, more data are required to inform the rational selection of therapeutic candidates for inclusion.

To identify drug candidates likely to be of benefit, two outstanding questions must first be answered: (i) is structural similarity between HTLV-1 and HIV-1 sufficient to allow for binding of existing reverse transcriptase inhibitors? If so, (ii) do any of these inhibitors bind with sufficient affinity and in the correct conformation to inhibit HTLV-1 infective spread at a tolerable dose? The crystal structures of HTLV-1 retroviral proteins have not been resolved which has limited conventional structure-based analyses [13]. To bring greater attention to this neglected pathogen, we have addressed the above questions using in silico homology modelling and machine learning to predict a structural model of the HTLV-1 reverse transcriptase, something that has not previously been achieved. Using this model, we have performed molecular docking experiments to provide a framework to identify which, if any, existing retroviral reverse transcriptase inhibitors with FDA approval could be candidates for clinical testing against HTLV-1.

## Results

**Fig. 1:**
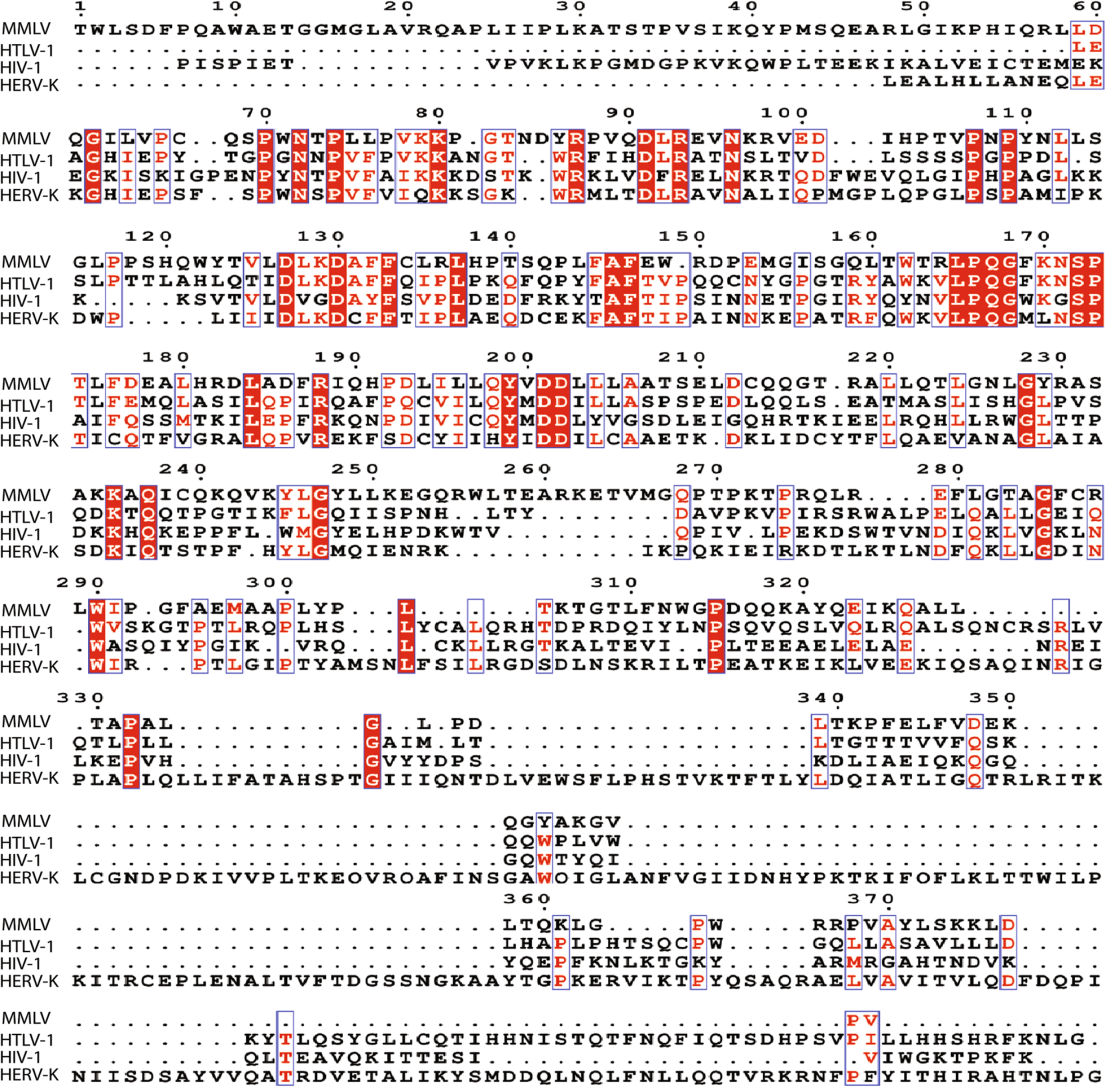
MMLV, HTLV-1, HIV-1, and HERV-K sequence alignment. Amino acid sequence alignment of MMLV, HTLV-1, HIV-1, and HERV-K. Amino acids conserved between all three viruses are highlighted in red with white text. For those residues considered for at least two viruses are outlined in blue with red text. Insertions are represented by a period

**Fig. 2:**
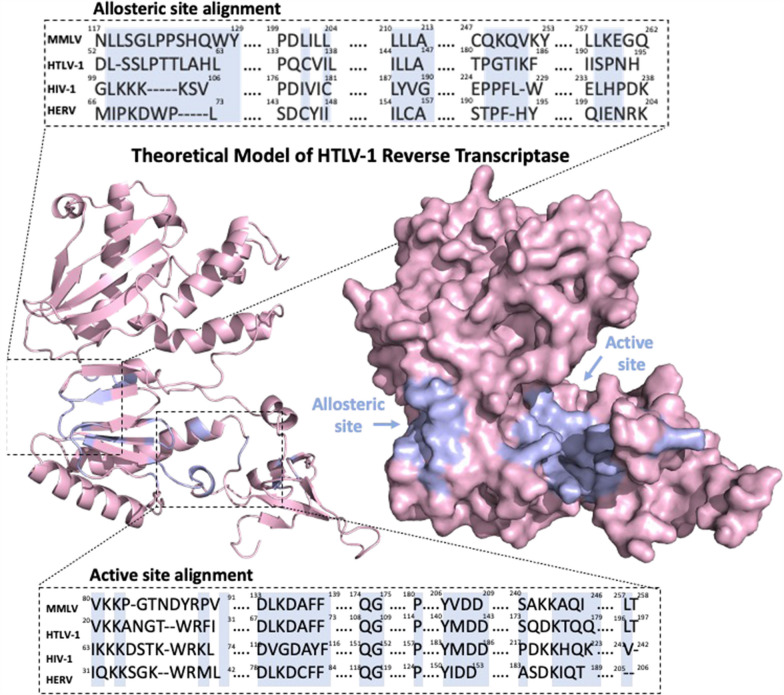
Rationalisation for theoretical model for HTLV-1 reverse transcriptase and molecular docking of reverse transcriptase inhibitors. **A** Cartoon representation of predicted HTLV-1 reverse transcriptase Alphafold2 model, and **B** its molecular surface. The predicted binding site of non-nucleoside reverse transcriptase inhibitors (NNRTIs) (allosteric site) and the binding site of nucleoside reverse transcriptase inhibitors (NRTIs) (active site) have been highlighted in purple. Amino acid sequence alignment for the active sites of HTLV-1 (NCIB: NC_001436), MMLV (PDBID:4MH8), HIV-1 (PDBID:1JLA), and HERV (PDBID:7SR6). Highlighted in purple are the amino acids defined for MMLV, HIV-1, and HERV (and predicted for HTLV-1) to interact with NNRTIs (above) and with NRTIs (below). Related to Additional file 1: Fig. S1

To address the above questions, it was first necessary to predict the structure of the HTLV-1 reverse transcriptase. Even among related species, DNA and amino acid sequences are often divergent. Despite this, protein structures tend to be highly conserved, presumably owing to the essential relationship between structure and function. By taking advantage of this, homology modelling can provide a theoretical prediction of a protein’s structure if the encoding DNA sequence is known and if crystal structure information is available for equivalent proteins of related species [14, 15]. The HIV-1 (protein databank identification number [PDBID]:1JLA), Moloney Murine Leukemia Virus (MMLV) (PDBID:4MH8), and Human Endogenous Retrovirus K (HERV-K) (PDBID:7SR6) retroviruses have been previously shown to share DNA and amino acid sequence similarity to HTLV-1 (NCIB: NC_001436) [16]. Based on previous annotations of HIV-1, MMLV, and HERV-K sequences, it was possible to infer within the HTLV-1 sequence, a 390 amino acid sequence (Gag-Pro-Pol amino acids 614–1004; annotated in NCIB:NC_001436) likely to contain all necessary domains to form the final reverse transcriptase structure. When comparing proteins, those with greater than 25% amino acid sequence similarity, usually take homologous 3D structures. Encouragingly, similarity was high between the identified HTLV-1 sequence and reverse transcriptases of HIV-1 (25%), MMLV (27%), and HERV-K (29%) (Fig. 1) [14–16]. This provided confidence that the inferred amino acid sequence was highly likely to be associated with the HTLV-1 reverse transcriptase. To model the HTLV-1 reverse transcriptase structure, the identified 390 amino acid sequence was then input into Alphafold2; a machine learning algorithm which incorporates sequence homology, structural homology, secondary structure prediction, with contact maps (a ‘fingerprint’ of amino acid interactions in a folded structure), and has been reported to make highly accurate predictions of thousands of protein structures [17]. Through this process, Alphafold2 was able to generate 5 theoretical models of HTLV-1 Reverse transcriptase (Fig. 2A and B). For further analyses, the model with the lowest predicted alignment error (PAE) score was used (Additional file 1: Fig. S1A). Further, this model demonstrated a typical resemblance to defined reverse transcriptases and had an obvious DNA binding pocket (Additional file 1: Fig. S1B). To determine the most energetically favourable conformation of the model and its proper molecular arrangement in 3D space, the structure was energy-minimized using GROMACS (Additional file 1: Fig. S1C).

**Fig. 3:**
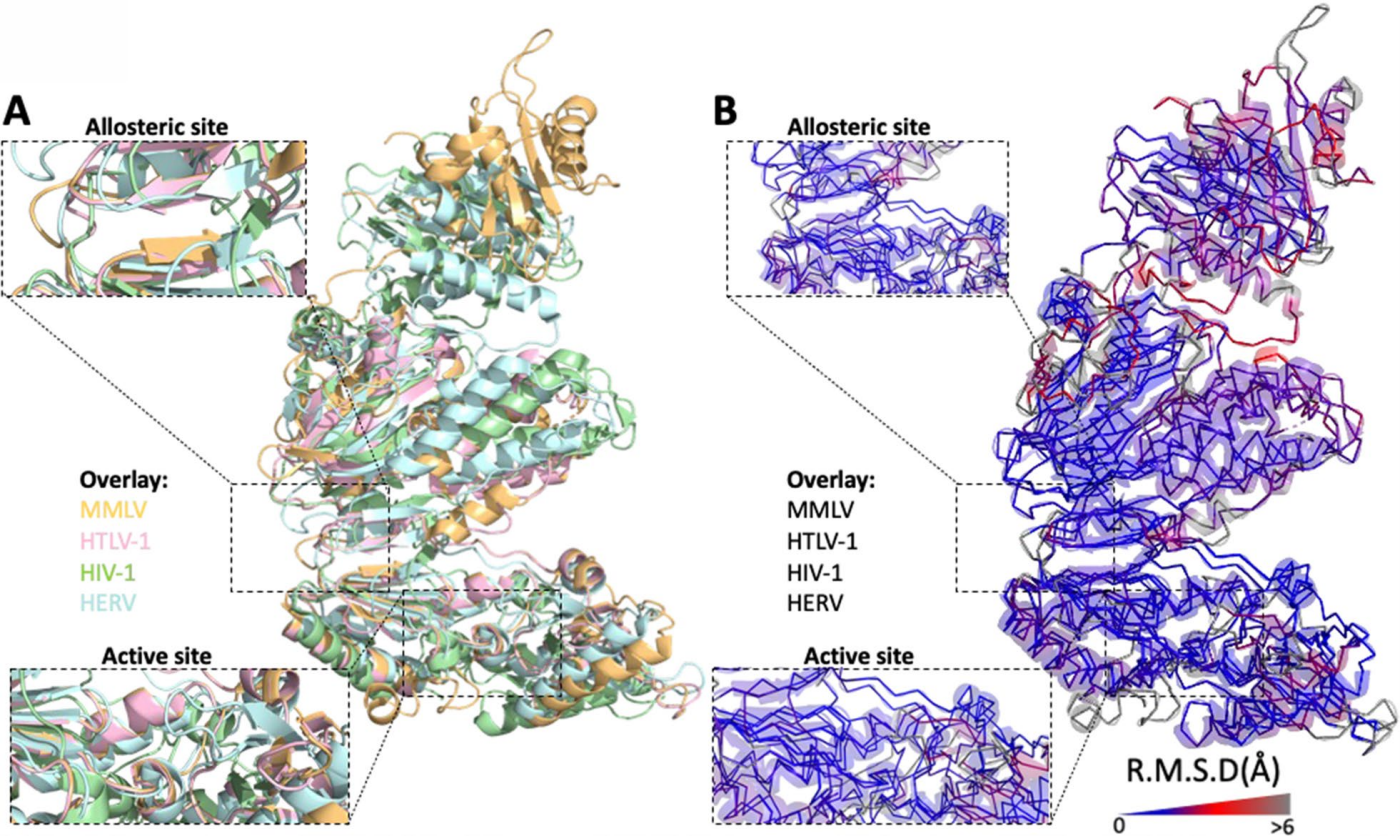
Reverse transcriptase structural comparison between MMLV, HTLV-1, HIV-1, and HERV-K. **A** Cartoon representation of HTLV-1 (pink), MMLV (orange), HERV (blue), and HIV-1 (green) reverse transcriptases (right). Inlays represent the active or allosteric site for each representation. **B** As for (A), ribbon diagram of backbone structural divergence measured as R.M.S.D. (Å) and depicted as blue (low) to grey (high) colour gradient (right). Inlays represent the active or allosteric site for each representation

To assess similarity between the predicted theoretical HTLV-1 model and those previously defined for MMLV (PDBID:4MH8), HERV-K (PDBID:7SR6), and HIV-1 (PDBID:1JLA), comparisons were made using root mean square deviation (R.M.S.D.); a commonly used quantitative measure of variation between superimposed atomic coordinates [18, 19]. Generally, R.M.S.D. values of < 3.5 Å suggest a high degree of similarity (i.e. low structural variance). The HTLV-1 reverse transcriptase model was found to be highly structurally similar to MMLV (R.M.S.D. 0.109 Å); however, some structural variation was seen when it was compared to either HIV-1 (R.M.S.D. 4.282 Å) or HERV-K (R.M.S.D. 3.936 Å). It is worth noting; however, that these variations were modest in comparison to structural variation between HIV-1 and MMLV (R.M.S.D. 10.225 Å) (Fig. 3A to B and Table 1).

**Table 1:** Structural alignment of different species root mean square derivation score (R.M.S.D.) and homology model comparison for either whole reverse transcriptase structure, nucleoside reverse transcriptase inhibitor (NRTIs) binding site (active site), or non-nucleoside reverse transcriptase inhibitor (NNRTIs) binding site (allosteric site)

	1jla	alpha	4mh8	7sr6
*Whole structure*				
1jla	100			
alpha	4.282	100		
4mh8	10.225	0.109	100	
7sr6	3.956	3.936	11.075	100
*Allosteric*				
1jla	100			
alpha	4.765	100		
4mh8	1.847	0.083	100	
7sr6	3.494	2.559	1.554	100
*Active*				
1jla	100			
alpha	5.03	100		
4mh8	2.563	1.3464	100	
7sr6	4.746	4.332	4.244	100

	Phyre^2^	alpha	Swiss	Mod
*Whole structure*				
Phyre^2^	100			
alpha	3.109	100		
Swiss	3.601	1.909	100	
Mod	3.853	2.081	2.488	100
*Allosteric site*				
Phyre^2^	100			
alpha	1.109	100		
Swiss	1.466	1.552	100	
Mod	1.782	1.407	1.611	100

On the basis of previous annotations of HIV-1, it was possible to identify two sites within the HTLV-1 reverse transcriptase model likely to bind inhibitors of reverse transcriptase. These were the allosteric site which is targeted by non-nucleoside reverse transcriptase inhibitors (NNRTIs) and the active site which directly interacts with DNA and is the target of nucleoside analogue reverse transcriptase inhibitors (NARTIs or NRTIs) (Fig. 2). Similar to the comparisons of the whole structures the HTLV-1 reverse transcriptase, when compared with MMLV, the active and allosteric sites showed minimal structural variation (R.M.S.D. allosteric site, 0.083 Å; active site, 1.346 Å). For comparisons between the HTLV-1 reverse transcriptase model active and allosteric sites and those of either HIV-1 (R.M.S.D. allosteric site, 4.765 Å; active site, 5.03 Å), or HERV-K (R.M.S.D. allosteric site, 2.559 Å; active site, 4.332 Å), structural variation was again seen (Fig. 3A to B and Table 1). These findings suggest that despite sharing many structural characteristics, some overall and domain-specific structural variation exists between the HTLV-1 reverse transcriptase and those of HIV-1 and HERV-K, and to a lesser extent MMLV.

**Fig. 4:**
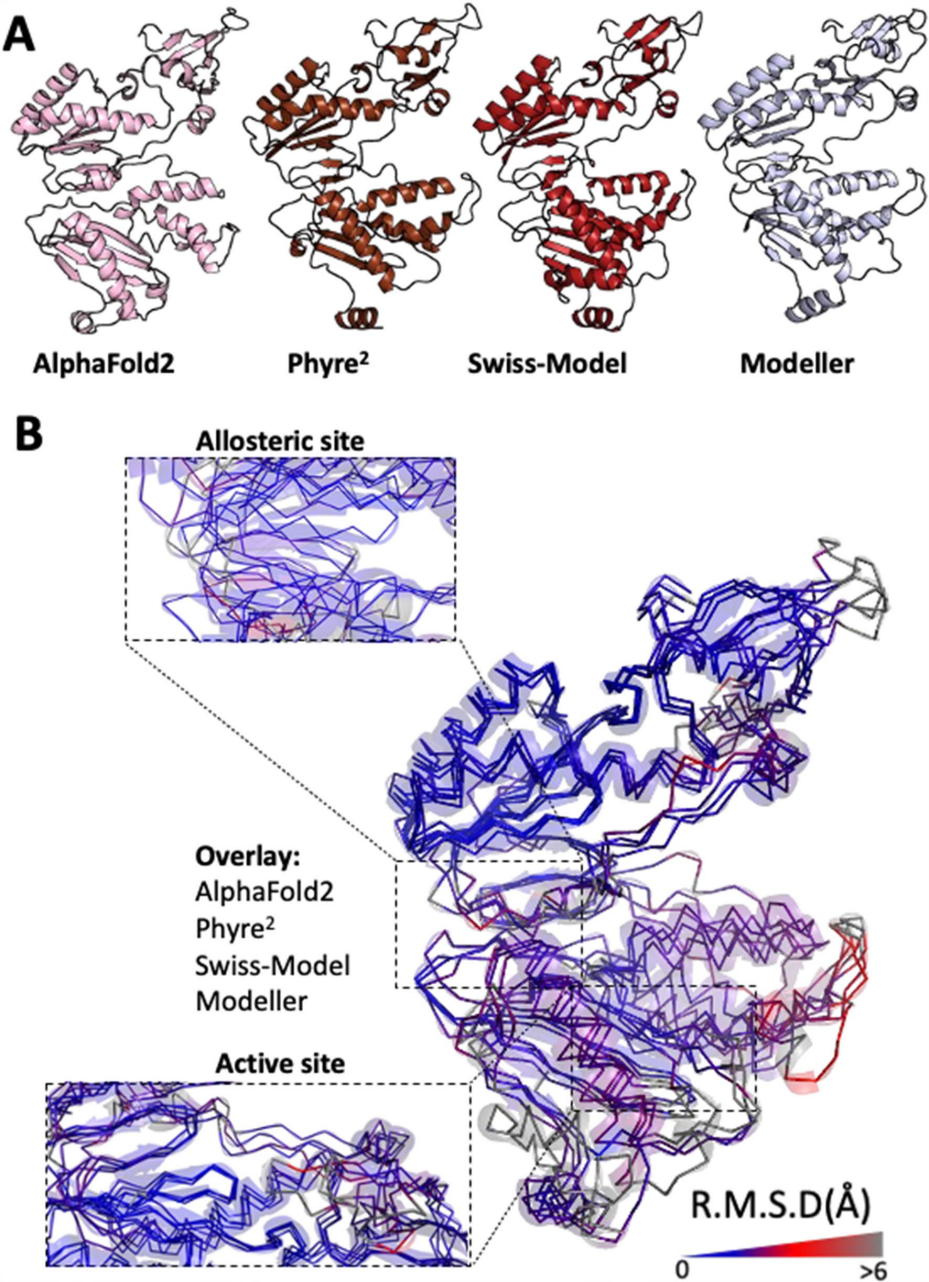
Modelling of theoretical HTLV-1 reverse transcriptase using alternative methods. **A** Cartoon representations of theoretical HTLV-1 reverse transcriptase modelled using Alphafold2, Phyre^2^, Swiss-Model, and Modeller. **B** As for (A), ribbon diagram of backbone structural divergence measured as R.M.S.D. (Å) and depicted as blue (low) to grey (high) colour gradient. Inlays represent the active or allosteric site for each representation

While advances in protein structural analysis are constantly being made, homology modelling can be an error-prone process, reliant on the assumptions and the data available to each software package. As such, orthogonal validation using crystal structure information is often important; however, when this information is unavailable, greater confidence in the predicted structure can be gained using separate methods [15, 20]. For example, using an identified amino acid sequence, it is possible to repeat structural modelling using separate software packages. From these separate models, structures identified to be generally similar are thought to be more likely representative of the protein’s native structure [15, 20]. For this, the inferred HTLV-1 reverse transcriptase amino acid sequence was input to three additional software packages: the Phyre^2^ protein folding web server [21], Modeller [22], and Swiss-Model, all of which build iterative models based on sequence and structural information (Fig. 4A) [23]. Structural variation between the overall models was minimal (R.M.S.D. 1.909–3.853 Å), and further improved for the allosteric and active sites (R.M.S.D. 1.109–1.782 Å) (Table 1); providing greater confidence in the likelihood that the identified HTLV-1 amino acid sequence is representative of the native structure of the reverse transcriptase (Fig. 4A to B) [18, 19]. Comparing the four predicted models, the greatest source of variation was introduced by the Modeller result, which by comparison, produced a model of HTLV-1 reverse transcriptase complexed with DNA, leading to larger shifts in secondary structure (Fig. 4A to B).

**Fig. 5:**
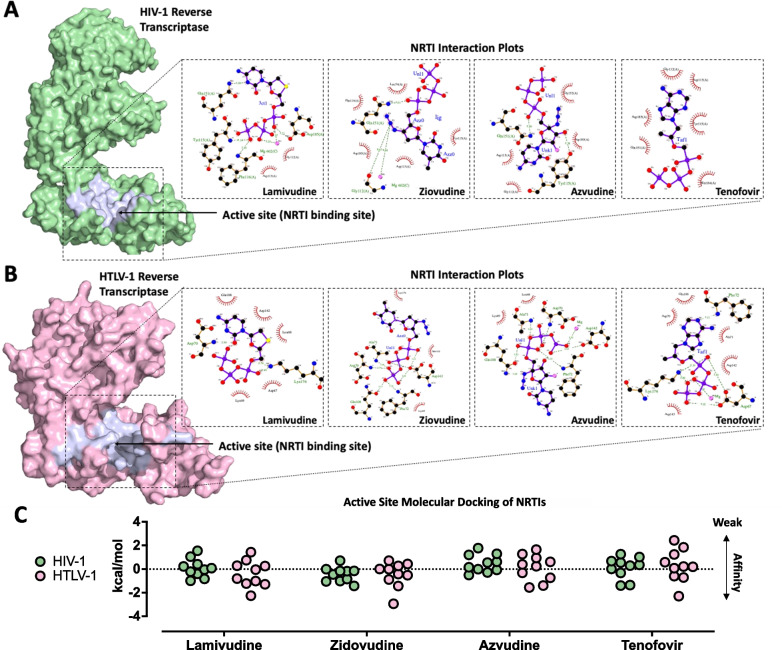
Molecular docking of reverse transcriptase inhibitors to HIV-1 and HTLV-1 reverse transcriptase. **A** Molecular surface diagram of HIV-1 reverse transcriptase with nucleoside reverse transcriptase inhibitor (NRTIs) binding site (active site) highlighted purple (left). Interaction plots of indicated NRTIs in the active site in their most energetically favourable conformation (1 of 10) (right). **B** Molecular surface diagram HTLV-1 reverse transcriptase with nucleoside reverse transcriptase inhibitor (NRTIs) binding site (active site) highlighted purple (left). Interaction plots of indicated NRTIs in the active site in their most energetically favourable conformation (1 of 10) (right). **C** Data summary of molecular docking testing 10 different conformations in either the HIV-1 reverse transcriptase or HTLV-1 reverse transcriptase. Related to Additional file 1: Fig. S2

Various in vitro studies, and a handful of clinical studies have suggested that some FDA-approved inhibitors of reverse transcriptase might have therapeutic activity against HTLV-1 [8]. As a cautionary note, HTLV-1 is known to behave unusually in vitro meaning that it can be challenging to interpret these findings, and clinical studies performed to date have primarily focused on individuals with severe ATLL or HAM-TSP, which might confound results [8]. To provide greater clarity and context to these previous findings, we therefore wanted to test whether a structural basis for binding of these therapies exists within the HTLV-1 reverse transcriptase model, especially given that many of these therapies were designed to inhibit the HIV-1 reverse transcriptase allosteric and active sites. To do this, we selected four NNRTIs (rilpivirine, doravirine, nevirapine, and dapivirine) and four NARTIs or NRTIs (tenofovir alafenamide, zidovudine, lamivudine, and azvudine) to test with in silico docking experiments. Encouragingly, the HTLV-1 reverse transcriptase model had a more apo-enzyme-like form (an open or unbound character) than that of the HIV-1 reverse transcriptase, making it an ideal candidate for molecular docking simulations. These were performed using Autodock 4 which is capable of simulating interactions of molecules in different conformations within a protein structure and in doing so, can calculate interaction-associated binding energies (values < 0 kcal/mol are favourable) [24]. Although other molecular docking programs exist, we chose Autodock 4 as it is able to handle molecule-ion interactions such as those which occur in the active site of the HTLV-1 reverse transcriptase model with Mg^2+^ for which we have simulated a single ion (Additional file 1: Fig. S2D). While two ions are important for reverse transcriptase catalytic activity, it is not yet clear whether these are essential for therapeutic binding (for example: PDBID:7DBN and PDBID:7AIF). As a control, we first tested molecular docking of each drug against the HIV-1 reverse transcriptase (PDBID:1JLA) (Additional file 1: Fig. S2A and C). NNRTIs were found to bind strongly in the allosteric site and each was tested in 10 different conformations providing binding energies ranging from − 9.74 to − 4.94 kcal/mol for doravirine, − 7.02 to − 1.49 kcal/mol for dapivirine, − 8.27 to − 4.39 kcal/mol for nevirapine, and − 2.77 to 13.08 kcal/mol for rilpivirine (Additional file 1: Fig. S2A and C). Before molecular docking of NRTIs was attempted, they needed to be converted to biologically active, phosphorylated prodrug metabolites [25]. NRTIs were also found to bind strongly to the HIV-1 reverse transcriptase active site with − 1.40 to 1.26 kcal/mol for tenofovir alafenamide, − 1.43 to 0.71 kcal/mol for zidovudine, − 1.00 to 1.55 kcal/mol for lamivudine, and − 0.51 to 1.77 kcal/mol for azvudine (Fig. 5A and C). In comparison with the HIV-1 reverse transcriptase allosteric site, the HTLV-1 reverse transcriptase allosteric site contained significantly more hydrophobic amino acids. Consequently, NNRTIs were unable to form hydrogen bonds in the allosteric site, yielding extremely poor binding affinities for 198.43 to 11.07 kcal/mol for doravirine, 75.58 to 6.69 kcal/mol for dapivirine, 27.15 to 15.38 kcal/mol for nevirapine, and 137.19 to 25.04 kcal/mol for rilpivirine (Additional file 1: Fig. S2B, C). This suggested that the NNRTIs tested might not have antiviral activity against HTLV-1. Surprisingly, despite R.M.S.D. differences between the HIV-1 and HTLV-1 reverse transcriptase active sites, interactions between NRTIs and the HTLV-1 reverse transcriptase active site were associated with improved binding energies, with − 2.3 to 2.43 kcal/mol for tenofovir alafenamide, − 2.94 to 0.71 kcal/mol for zidovudine, − 2.26 to 1.43 kcal/mol for lamivudine, and − 1.56 to 1.65 kcal/mol for azvudine (Fig. 5A–C). However, based on comparisons (data not shown) between the HIV-1 control used in this study (PDBID:1JLA) which is known to be in an open conformation (apoenzyme form, catalytically inactive), and an HIV-1 RT structure known to be in a closed conformation (PDBID:4PQU) (holoenzyme, catalytically active), and our HTLV-1 RT structure, it can be inferred that our HTLV-1 RT model is in a catalytically inactive, open conformation. Currently, it is not possible using existing non-template-based protein folding methods to produce a model complexed with nucleic acid to represent a closed conformation, and that NRTI binding strengths might be further improved in this context.

## Discussion

In this study we have used homology modelling and machine learning to develop a reasonable approximation of the HTLV-1 reverse transcriptase and used molecular docking to understand its binding interactions with FDA-approved inhibitors of reverse transcriptase. Together, these data suggest that chemical and structural dissimilarity between the reverse transcriptases of HIV-1 and HTLV-1 likely limits the efficient binding, and in turn potential for therapeutic efficacy of NNRTIs. Few if any studies have evaluated the capacity of NNRTIS such as rilpivirine, doravirine, nevirapine, and dapivirine to inhibit the HTLV-1 reverse transcriptase. Given their specificity for HIV-1, this is perhaps unsurprising. In fact, NNRTIs are incapable of inhibiting the HIV-2 reverse transcriptase (PDBID: 1MU2) which has a sequence similarity of 42% to that HIV-1, which is about twice as great as that between HIV-1 and HTLV-1 [26]. By contrast, the structural properties of the reverse transcriptase demonstrated clear and efficient binding to NRTIs which exceeded that of binding to the HIV-1 reverse transcriptase. Importantly, these two proteins’ active sites share a conserved YMDD motif which is important for Mg2^+^ coordination to triphosphates in each of the therapies tested, while differences in binding strength between the HTLV-1 and HIV-1 reverse transcriptase active sites occurred due to differing chemical properties of surrounding amino acids which hold each of the therapies in place but not their triphosphates. These findings are important and support various preclinical and clinical studies performed to date. For example, zidovudine (AZT) has been demonstrated using in vitro and in vivo studies to be associated with decreased proviral load suggesting a capacity to limit infective spread [7, 27]. Although clinical studies investigating the role of AZT in treatment of HTLV-1 infection do not appear to have been performed, for treatment of HTLV-1-associated ATLL, AZT in combination with interferon *α* (IFN*α*) is currently recommended for treatment of symptomatic smouldering, unfavourable chronic, lymphoma (including extranodal primary cutaneous variant), and for acute disease with non-bulky tumor lesions [28]. From in vitro studies, it is clear that lamivudine has some capacity to protect lymphocytes from infection, however a methionine-to-valine substitution in the conserved motif of the HTLV-1 RT, tyrosine (Y)-methionine (M)-aspartic acid (D)-aspartic acid (D) (YMDD), has been shown to confer resistance in a similar way to how the M184V substitution which confers lamivudine resistance in HIV-1 RT [29]. In a small clinical study of patients with HAM-TSP lamivudine treatment coincided with a temporary decrease in circulating proviral load which rebounded back to baseline within 24 weeks of treatment [5]. Tenofovir has demonstrated in vitro inhibition of HTLV-1 reverse transcriptase; however, a small study in which daily treatment with 254 mg of tenofovir for a mean of 8.7 (± 2.3) months was not associated with a reduction in proviral load [30]. It is not clear whether azvudine has been tested for antiviral activity against HTLV-1.

Although clinical studies performed to date suggest that NRTIs have modest therapeutic benefit against HTLV-1, it is very important to recognise that the studies performed to date, have been on chronically infected individuals or those with severe ATLL or HAM/TSP. In chronically infected individuals, HTLV-1 viral activity is relatively quiescent, and reverse transcriptase-mediated infective spread contributes minimally to viral propagation, instead the proviral load is maintained by clonal proliferation. This suggests that targeting the HTLV-1 reverse transcriptase to treat chronically infected individuals might have limited efficacy [27]. By contrast, the acute phase which occurs in the months following infection is strongly associated with reverse transcriptase-mediated infective spread, meaning that this is the period during which an individual would be most likely sensitive to reverse transcriptase inhibition. This is the rationale for testing of these therapies using pre- and post-exposure prophylaxis regimens [27].

It is important to note that drugs targeting other retroviral proteins such as integrases and proteases do exist and are FDA-approved for various indications. A recent study used in vitro assays to identify HTLV-1 integrase inhibitors and found several candidates with potential activity against HTLV-1. Although these assays identified several drugs, the following in silico docking of these was only used to provide qualitative structural insight. For metal ion coordinated, proteins such as integrases, it is currently difficult to use in silico approaches to derive quantitative molecular docking results as ion coordination presents challenges for existing software packages [30, 31].

A limitation of our study was although the HTLV-1 reverse transcriptase can probably function as a monomer, it is most likely to be heterodimer (p51 and p66) in situ [32, 33]. While generating such a structure might improve the overall structural accuracy of our model, there are currently limits to creating structures such as these with Alphafold2. Nonetheless, we have modelled the drug targets themselves in HTLV-1 p66 which is the subunit responsible for the reverse transcription reaction [33]. Modelling a heterodimeric structure is unlikely to have affected the simulation results and therapeutic binding kinetics presented here. By modelling both subunits of the heterodimer, but focusing on one active site would be somewhat redundant as this molecular docking method does not account for conformational changes. A more highly advanced iterative, in silico workflow could be applied involving molecular dynamics of the heterodimer; however the conformational changes in the active and allosteric sites simulated would not occur due binding of the therapies, but as a consequence of structural equilibration. Therefore, the approach presented here, while pragmatic, should reflect the binding trends of the HTLV-1 active and allosteric sites. For our control docking simulations, we used only one model of HIV-1 RT (PDBID:1JLA). Although there are other models now available (PDBID:4PQU) the amino acid sequence similarity between the p66 subunits of each structure are extremely high (96.98%), nonetheless it is a limitation that just one control structure was used.

HTLV-1 remains a neglected area of basic and clinical research. Following decades of intensive research on the pathogenesis of HIV-1, the tools now exist to understand the biology of HTLV-1 and for rational therapeutic development to take place. In this study, we aimed to understand whether a structural basis for binding to inhibitors of reverse transcriptase exists within the HTLV-1 reverse transcriptase. Limited by an unresolved protein structure, we developed and tested a theoretical model of HTLV-1 reverse transcriptase based on sequence alignment, homology modelling, and machine learning. Using this model, we identified that NRTIs such as tenofovir alafenamide, zidovudine, lamivudine, and azvudine are likely capable of binding and inhibition of the HTLV-1 reverse transcriptase.

## Methods

### Sequence alignment and homology modelling

To construct a viable sequence to use for de novo folding, homology modelling, and sequence alignment was done as a preliminary step to gauge an appropriate enzyme size with respect to number of amino acids. Using CLC Main Workbench (QIAGEN), the amino acid sequence of HTLV-1 Gag-Pro-Pol was aligned to that of HIV-1 reverse transcriptase (PDBID:1JLA), HERV-K reverse transcriptase (PDBID:7SR6), and MMLV reverse transcriptase (PDBID:4MH8) to look for conservation in sequence and infer the sequence for HTLV-1 reverse transcriptase. Using the pairwise analysis tool in CLC, a Point Accepted Mutation matrix (PAM) was constructed via the Dayhoff and Schwartz method (Dayhoff and Schwartz—Atlas of protein sequence and structure vol 3 of 5) to calculate the level of homology between proteins. In addition to sequence alignment was performed to enable, active site and allosteric site identification by structural alignment of HIV-1, HERV-K, and MMLV reverse transcriptases complexed with inhibitors where information was available: Allosteric site (PDBID; 1JLA, 1JLC, 1JEK),and Active site (PDBID; 5TXM, 7RS6, 4HKQ).

### De novo* folding*

The 390 amino acid sequence inferred to encode the HTLV-1 reverse transcriptase was input into the publicly available Alphafold2, Modeller, Swiss-Model, and Phyre^2^ web servers to produce structures as previously described [17, 18, 21, 23]. Related to the Modeller result, structural alignment was also performed with HERV (PDBID:7SR6). In situ, the reverse transcriptase forms a heterodimer with one of the monomers split into four different domains, the finger, palm, thumb, and Rnase H domains classified as the p66 subunit. The other subunit (p51) is missing the Rnase H domain and is folded differently; however, because the active and allosteric sites are exclusively located in the p66 subunit, the sequence associated with p66 was used for modelling of the HTLV-1 reverse transcriptase.

### Energy minimisation

Energy minimisation was performed to relax the initial backbone conformation of the final reverse transcriptase structure and the active site. Using the GROMACS (5.31) simulation package a two-step energy minimization was done for a total of 2000 steps, with the first 1000 steps using the steepest decent method, followed by a further 1000 steps of conjugate gradient algorithm [34]. This was performed in explicit water using the tip4p water while interatomic interactions were modelled using AMBER force field (ffSB14) [35, 36].

### Molecular docking

The Alphafold2 structure was tested against a series of 8 different drugs in an in silico docking experiment, 4 NNRTIs in the allosteric site and 4 NRTI’s in the active site. These were carried out using the freely available AutoDock4 which is part of the Autodock Tools, which is a suite of programs used to prepare a protein and its corresponding drug target for Autodock4. These programs include Mgltools, PyMolecular View and Racoon [37]. Each drug target was localised to the predetermined interaction site, using the HIV-1 reverse transcriptase (PDBID:1JLA) and HERV-K (PDBID:7SR6) as a reference, with each docking experiment run through a series of 10 conformations via the generic search algorithm. The Mg^2+^ parameters were handled using an external parameter file, AD4_parameters.dat (https://autodock.scripps.edu/how-to-add-new-atom-types-to-the-autodock-force-field/).

### Ligand protein interactions

Visualisation of the ligands (NNRTI and NRTIs) and their associated interactions in the active and allosteric binding sites were visualised using Ligplot+. Using the best or lowest energy structure of the ligand in the binding pocket, interactions were visualised as either hydrogen bonds, dotted green lines or Van der Waals interactions, red semi-circles. Atoms were coloured using the CPK colouring method, while bonds were coloured purple. Confirmations with either more hydrogen bonds (green dotted lines) or more Van der Waals interactions, suggested a better fit (lower interaction energy and better ligand–protein interaction) at the binding site.

### Analysis

PyMol Molecular Graphics System Version 1.2r3pre (Schrödinger, LLC) was used to visualise folded protein structures and docking results. To compare target structures HIV-1, HERV-K, and MMLV to the HTLV-1 structure, the alignment tool was used and reported as the deviation of the backbone from the target structure (HIV-1) and reported in R.M.S.D. in Å. Blue to red scale was used to represent an approximation of backbone deviation using the colourbyrmsd plug-in.

### Supplementary Information

**Additional file 1: Fig. S1. A **Plots of predicted alignment error (PAE) for 5 different HTLV-1 reverse transcriptase models generated using Alphafold2, the model with the lowest PAE (rank_1) was used. **B** Cartoon representation of the Alphafold2 model theoretically complexed with DNA (green) using HERV-K (PDBID:7SR6) and MMLV (PDBID:4MH8) models. **C** Cartoon representation of theoretical HTLV-1 reverse transcriptase (Alphafold2) (light pink) overlayed with energy-minimized structure (GROMACS 5.3.1) (dark pink) (left). Backbone structural divergence measured as R.M.S.D. (Å) and depicted as blue (low) to grey (high) colour gradient (right). Inlay represents the active site (predicted site of reverse transcriptase inhibitor binding) amino acids for the non-energy minimized (pink) and energy minimized (light pink) structures. **Fig. S2. A** Molecular surface diagram of HIV-1 reverse transcriptase with non-nucleoside reverse transcriptase inhibitor (NNRTIs) binding site (allosteric site) highlighted purple (left). Interaction plots of indicated NNRTIs in the active site in their most energetically favourable conformation (1 of 10) (right). **B** Molecular surface diagram of HTLV-1 reverse transcriptase with non-nucleoside reverse transcriptase inhibitor (NRTIs) binding site (allosteric site) highlighted purple (left). **C** Data summary of molecular docking testing 10 different conformations in either the HIV-1 reverse transcriptase or HTLV-1 reverse transcriptase. **D** Molecular surface diagram of HTLV-1 reverse transcriptase with nucleoside reverse transcriptase inhibitor (NRTIs) binding site (active site) highlighted purple (left). Inlay of Mg2+ coordination within the active site.

## Data Availability

All data are available on request to the corresponding author.
