# Supplementary material for: HTLV-1 reverse transcriptase homology model provides structural basis for sensitivity to existing nucleoside/nucleotide reverse transcriptase inhibitors
Source: Virol J. 2024 Jan 10;21:14. doi: 10.1186/s12985-024-02288-z (PMC10782711; doi:10.1186/s12985-024-02288-z)
Supplement: Supplementary file 1 — Additional file 1: Fig. S1. A Plots of predicted alignment error (PAE) for 5 different HTLV-1 reverse transcriptase models generated using Alphafold2, the model with the lowest PAE (rank_1) was used. B Cartoon representation of the Alphafold2 model theoretically complexed with DNA (green) using HERV-K (PDBID:7SR6) and MMLV (PDBID:4MH8) models. C Cartoon representation of theoretical HTLV-1 reverse transcriptase (Alphafold2) (light pink) overlayed with energy-minimized structure (GROMACS 5.3.1) (dark pink) (left). Backbone structural divergence measured as R.M.S.D. (Å) and depicted as blue (low) to grey (high) colour gradient (right). Inlay represents the active site (predicted site of reverse transcriptase inhibitor binding) amino acids for the non-energy minimized (pink) and energy minimized (light pink) structures. Fig. S2. A Molecular surface diagram of HIV-1 reverse transcriptase with non-nucleoside reverse transcriptase inhibitor (NNRTIs) binding site (allosteric site) highlighted purple (left). Interaction plots of indicated NNRTIs in the active site in their most energetically favourable conformation (1 of 10) (right). B Molecular surface diagram of HTLV-1 reverse transcriptase with non-nucleoside reverse transcriptase inhibitor (NRTIs) binding site (allosteric site) highlighted purple (left). C Data summary of molecular docking testing 10 different conformations in either the HIV-1 reverse transcriptase or HTLV-1 reverse transcriptase. D Molecular surface diagram of HTLV-1 reverse transcriptase with nucleoside reverse transcriptase inhibitor (NRTIs) binding site (active site) highlighted purple (left). Inlay of Mg2+ coordination within the active site. [file 12985_2024_2288_MOESM1_ESM.docx]

**Supplemental data**

**Title**: HTLV-1 reverse transcriptase homology model provides structural basis for sensitivity to existing nucleoside/nucleotide reverse transcriptase inhibitors

**Authors**: Nicolas Tardiota, Noushin Jaberolansar, Julia A. Lackenby, Keith J. Chappell, and Jake S. O’Donnell.

**Figure S1. (A)** Plots of predicted alignment error (PAE) for 5 different HTLV-1 reverse transcriptase models generated using Alphafold2, the model with the lowest PAE (rank_1) was used. (B) Cartoon representation of the Alphafold2 model theoretically complexed with DNA (green) using HERV-K (PDBID:7SR6) and MMLV (PDBID:4MH8) models. **(C)** Cartoon representation of theoretical HTLV-1 reverse transcriptase (Alphafold2) (light pink) overlayed with energy-minimized structure (GROMACS 5.3.1) (dark pink) (left). Backbone structural divergence measured as R.M.S.D. (Å) and depicted as blue (low) to grey (high) colour gradient (right). Inlay represents the active site (predicted site of reverse transcriptase inhibitor binding) amino acids for the non-energy minimized (pink) and energy minimized (light pink) structures.

**Figure S2. (A)** Molecular surface diagram of HIV-1 reverse transcriptase with non-nucleoside reverse transcriptase inhibitor (NNRTIs) binding site (allosteric site) highlighted purple (left). Interaction plots of indicated NNRTIs in the active site in their most energetically favourable conformation (1 of 10) (right). **(B)** Molecular surface diagram of HTLV-1 reverse transcriptase with non-nucleoside reverse transcriptase inhibitor (NRTIs) binding site (allosteric site) highlighted purple (left). **(C)** Data summary of molecular docking testing 10 different conformations in either the HIV-1 reverse transcriptase or HTLV-1 reverse transcriptase. **(D)** Molecular surface diagram of HTLV-1 reverse transcriptase with nucleoside reverse transcriptase inhibitor (NRTIs) binding site (active site) highlighted purple (left). Inlay of Mg^2+^ coordination within the active site.

**
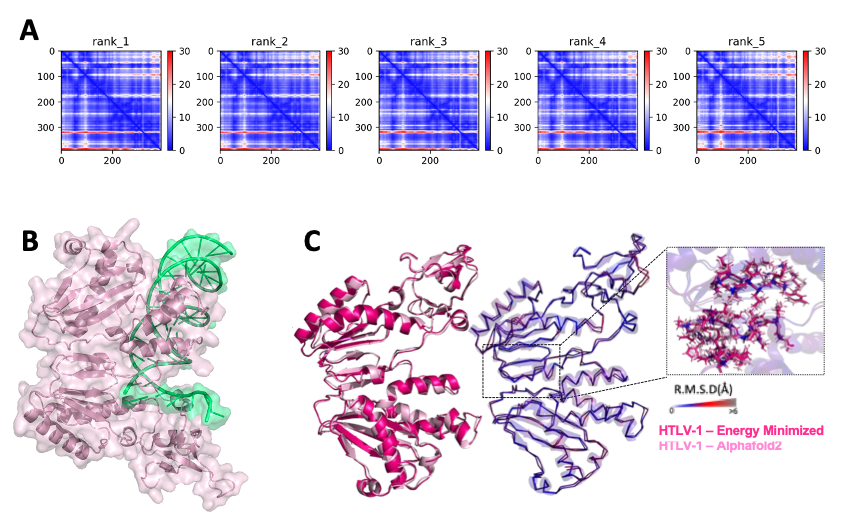
**

**Figure S1**

**
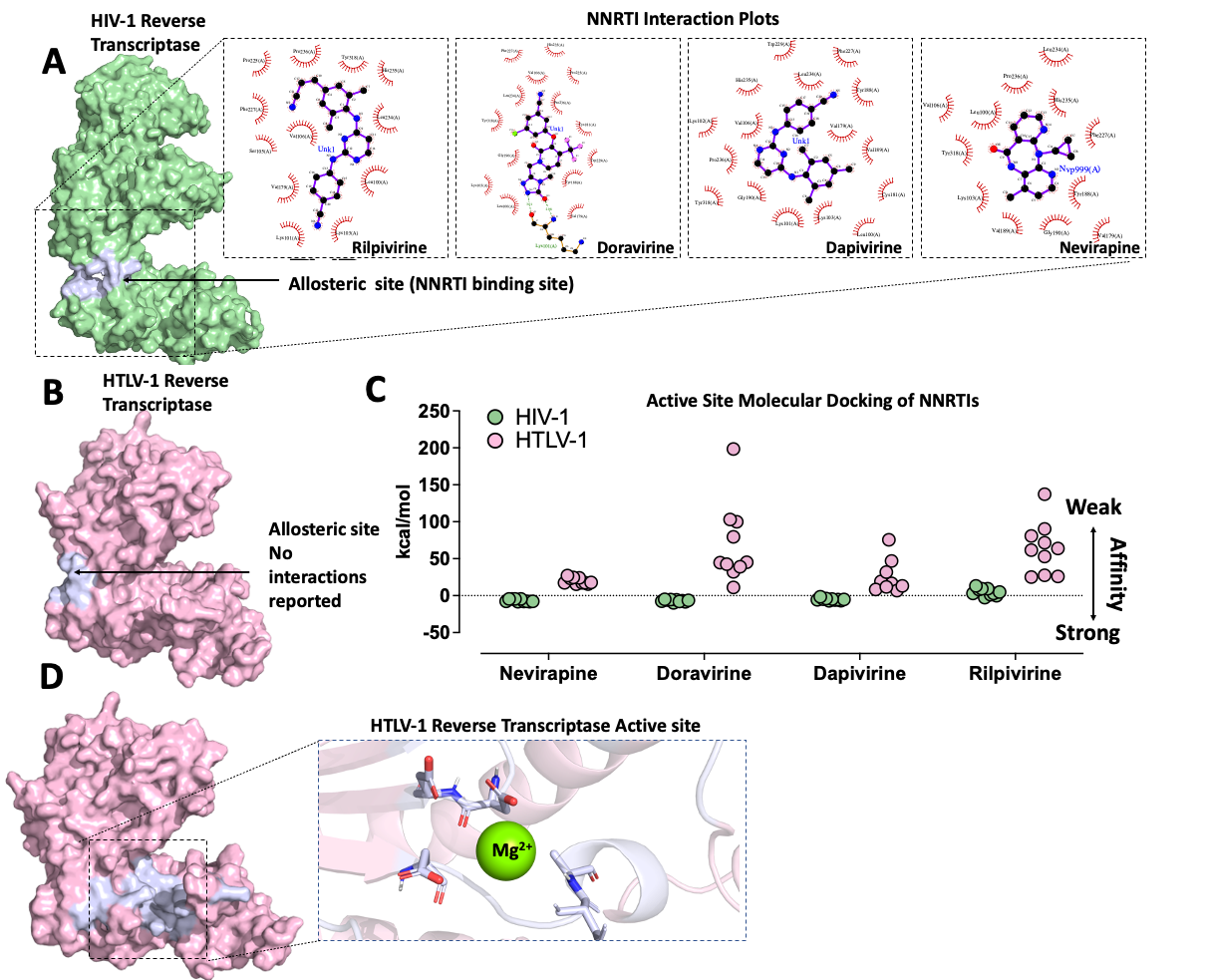
**

**Figure S2**
